# Steigende Inzidenzen bei Mastoidektomien im Kindesalter: Folge geringeren Antibiotikaeinsatzes oder eine COVID-19-Spätfolge?

**DOI:** 10.1007/s00106-024-01435-w

**Published:** 2024-03-01

**Authors:** Thomas Gehrke, Agmal Scherzad

**Affiliations:** 1 Klinik und Poliklinik für Hals‑, Nasen- und Ohrenkrankheiten, plastische und ästhetische Operationen Universitätsklinik, Universitätsklinikum Würzburg, Josef-Schneider-Str. 11, 97080 Würzburg, Deutschland; 2 Klinik für Hals-Nasen-Ohrenheilkunde, Universitätsklinikum Würzburg, Josef-Schneider-Str. 11, 97080 Würzburg, Deutschland

**Keywords:** Mastoiditis, Otologische chirurgische Verfahren, Infektiöse Knochenerkrankungen, Otitis media, Mittelohrentzündung, Mastoiditis, Otologic surgical procedures, Infectious bone diseases, Otitis media, Middle ear inflammation

## Abstract

**Hintergrund:**

In den Jahren 2022 und 2023 wurde eine steigende Anzahl an Mastoiditiden bei Kindern und damit auch ein Anstieg der Mastoidektomien bei Kindern beobachtet.

**Ziel der Arbeit:**

Ziel der vorliegenden Arbeit war es, den Anstieg der Anzahl an Patienten mit Mastoiditis und folgender Mastoidektomie zu analysieren, Korrelationen mit vorheriger Antibiotikatherapie und COVID-Infektionen zu untersuchen und einen Überblick über Keimspektrum, Krankheitsverlauf und Therapie zu geben.

**Material und Methoden:**

Dazu wurde eine retrospektive Analyse aller seit 2012 behandelten Patienten mit Mastoiditis durchgeführt, bei denen eine Mastoidektomie durchgeführt werden musste. Evaluiert wurden Art und Dauer vorangegangener Symptome und einer vorherigen Antibiotikatherapie, Diagnostik und Krankheitsverlauf sowie Keimspektrum, Dauer des Krankenhausaufenthalts und Komplikationen.

**Ergebnisse:**

Es wurde ein hoch signifikanter Anstieg an Mastoiditiden und somit auch an Mastoidektomien ab 2022 gezeigt. Weder Keimspektrum noch Krankheitsverlauf oder Komplikationsrate unterschieden sich von den vorherigen Jahren mit niedrigerer Inzidenz. Eher konnte eine Zunahme bereits ambulant antibiotisch vortherapierter Patienten gezeigt werden. Etwa die Hälfte der seit Herbst 2022 erkrankten Patienten hatte eine positive COVID-Anamnese. Adenoidhyperplasie spielte ursächlich eine deutlich geringere Rolle als in den Jahren zuvor.

**Schlussfolgerung:**

Eine Korrelation zu reduzierter ambulanter Antibiotikagabe erscheint bei in diesem Kollektiv eher steigender Anzahl somit kausal als unwahrscheinlich. Ob ein Zusammenhang mit einer durchgemachten COVID-Infektion besteht, kann aufgrund der hohen Dunkelziffer bei asymptomatischen und nicht nachgewiesenen Fällen nicht abschließend beurteilt werden.

## Abstract (en)

**Background:**

An increasing number of pediatric patients with mastoiditis and a consequent increase in mastoidectomy rates was noted in 2022 and 2023.

**Objective:**

This study aimed to analyze the increase in the number of children presenting with mastoiditis and subsequent mastoidectomy, to assess correlations with prior antibiotic treatment or COVID-19 infection, and to provide an overview of involved pathogens, treatment, and disease course.

**Materials and methods:**

A retrospective analysis of all patients with mastoidectomy since 2012 was conducted. Data collected comprised type and duration symptoms, prior antibiotic therapy, diagnostic tests and disease course, causal pathogens, length of hospitalization, and complications.

**Results:**

A highly significant increase in mastoidectomies in children could be demonstrated from 2022. Neither the pathogens involved nor the course of disease or complications showed differences. An increase in the number of patients with prior outpatient antibiotic therapy could be shown. About a half of the patients becoming ill after fall 2022 had a positive history of COVID. Hyperplasia of adenoid tissue was a far less frequent causal mechanism than in the years before COVID.

**Conclusion:**

No clear correlation with reduced outpatient antibiotic therapy could be found. Whether there exists an association with prior COVID infection cannot be judged at this time, due to the high number of asymptomatic and therefore unknown COVID infections.

Die Mastoidektomie stellt bei nicht auf Antibiotikagabe regredierter Mastoiditis weiterhin die Therapie der Wahl dar, sowohl bei Kindern als auch bei Erwachsenen. Innerhalb der letzten 2 Jahre konnte ein deutlicher Anstieg an kindlichen Mastoidektomien bemerkt werden, bei gleichzeitig im Wesentlichen gleichbleibender Anzahl an Mastoidektomien bei Erwachsenen. Dies wirft die Frage nach einer Ursache hierfür auf. Als mögliche Ursachen erschienen ein reduzierter Antibiotikagebrauch bei vorangegangener Otitis media sowie ein Zusammenhang mit einer COVID-Infektion denkbar.

## Akute Otitis media und Mastoiditis

Die akute Otitis media (AOM) ist weltweit die häufigste Diagnose und trifft gehäuft im Kleinkindalter auf. Die Daten der Centers for Disease Control and Prevention (CDC) zeigen eine Zunahme der AOM-Prävalenz in den Vereinigten Staaten von 150 %. Darüber hinaus konnte festgestellt werden, das bis zum zweiten Lebensjahr bereits 70 % der Kinder mindestens eine AOM-Episode durchgemacht haben [1]. Aus weiteren Untersuchungen geht hervor, dass AOM die Hauptursache für die Verschreibung von empirischen Antibiotika darstellt [2]. Die Erkrankung ist durch eine adäquate und zügige Behandlung i. d. R. gut therapierbar. Die deutsche Gesellschaft für Pädiatrische Infektiologie empfiehlt in der seit März 2022 aktualisierten Auflage die symptomatische Therapie einer AOM (https://dgpi.de). Eine Antibiotikatherapie wird hingegen nur bei Säuglingen unter 6 Lebensmonaten, schwerer oder komplikativer AOM, protrahierter AOM (mehr als 48–72 h Dauer) und bei relevanten Grunderkrankungen empfohlen. Die Diagnose der AOM wird klinisch durch die Ohrmikroskopie gestellt. Zu den Komplikationen der AOM gehört u. a. die Mastoiditis. Sie ist eine schwere bakterielle Infektion des Felsenbeins, welche als Folge einer akuten, seltener einer chronischen, Mittelohrentzündung auftreten kann. Vor der Antibiotika-Ära trat eine akute Mastoiditis in 1 von 5 Fällen einer akuten Otitis media auf [3]. Durch die rechtzeitige Antibiotikatherapie und die frühzeitige Behandlung der Tubenventilationsstörung in Form einer Adenotomie und Parazentese hat sich die Rate der akuten Mastoiditiden deutlich reduziert. Dennoch werden weiterhin regelmäßig Kinder mit einer akuten Mastoiditis stationär behandelt. Die Inzidenz der Mastoiditis liegt zwischen 1,2–6,2 pro 100.000 Kinder (0–14 Jahre) pro Jahr [4, 5]. Zu den wichtigsten Erregern gehören *Streptococcus pneumoniae* und *pyogenes*, gefolgt von *Haemophilus influenzae* und *Staphylococcus aureus*. Die Therapie der Mastoiditis kann sowohl konservativ als auch chirurgisch erfolgen. Die Therapie der Wahl ist von verschiedenen Aspekten wie Ausdehnung der Entzündung oder Begleitkomplikationen abhängig.

Die Inzidenzen vieler infektassoziierter Erkrankungen haben sich während der COVID-Pandemie verändert. Während der Pandemie wurden weltweit strenge Maßnahmen ergriffen, um die SARS-CoV-2-Infektionsraten zu senken. Zu diesen Maßnahmen gehörten u. a. die Schließung von Kindertagesstätten und Schulen, die Einführung von Homeoffice, die Reduzierung von Sitzgelegenheiten in Innenräumen, Reduktion von Sozialkontakten und die obligatorische Verwendung von Schutzmasken. Diese Maßnahmen führten einerseits zu einem Rückgang der Corona-Infektionen, andererseits aber auch zu einem Inzidenzabfall der nicht-Corona-assoziierten Infektionskrankheiten. In einer Studie von Tanislav und Kostev wurde die Häufigkeit von Infektionen der Atemwege und des Magen-Darm-Trakts während der COVID-Pandemie untersucht. Dabei wurde ein deutlicher Rückgang der Krankheiten der oberen Atemwege und der Darminfektionen festgestellt [6]. Die Studie zeigte auch einen signifikanten Rückgang der Influenzapneumonie um bis zu 90 % und der akuten Sinusitis um bis zu 66 % in der pädiatrischen Patientenpopulation. Diese Lockdown-Maßnahmen führten sogar zu einem drastischen Rückgang der Infektionen mit dem respiratorischen Synzytial-Virus (RSV) während der Hochsaison der Infektion bei Kindern. Diese Beobachtungen wurden durch die Studie von Kuitunen et al. untermauert. Sie analysierten die unmittelbaren Auswirkungen des Lockdowns auf die Inzidenz von Influenza A, Influenza B und dem RSV bei Kindern in Finnland [7]. Die Studie zeigte einen signifikanten Rückgang der Infektionen und einen damit verbundenen signifikanten Rückgang der Konsultationen in den pädiatrischen Notaufnahmen. Diese Beobachtungen wurden auch von Kruizinga et al. gemacht. Im Rahmen dieser retrospektiven Analyse stellten sie jedoch fest, dass die Zahl der Besuche in der pädiatrischen Notaufnahme nach dem Ende des Lockdowns wieder stark zunahm [8].

Ziel der vorliegenden monozentrischen retrospektiven Studie ist es, die gestiegene Anzahl der notwendigen Mastoidektomien bei Kindern seit 2022 zu analysieren und Rückschlüsse auf mögliche Ursachen zu ziehen.

## Material und Methoden

Es handelt sich um eine monozentrische retrospektive Datenanalyse. In diese wurden alle Patienten bis 14 Jahre eingeschlossen, bei denen zwischen dem 01.01.2012 und dem 31.06.2023 in der HNO-Universitätsklinik Würzburg eine Mastoidektomie bei akuter Mastoiditis durchgeführt wurde. Die Identifikation der Patienten erfolgte anhand des OPS-Codes (Operationen- und Prozedurenschlüssel) für Mastoidektomien über das klinikeigene digitale Betriebssystem. Die Indikation zur Mastoidektomie blieb während des gesamten Zeitraums unverändert und wurde gestellt in allen Fällen mit ausbleibender Besserung oder Symptomverschlechterung unter angemessener antibiotischer i.v.-Therapie oder bei Vorliegen von Komplikationen der Mastoiditis wie Sinusvenenthrombose, Subperiostalabszess oder intrakranieller Abszedierung. An präklinischen Daten wurden die Art und die Dauer bisheriger ohrbezogener Symptome, die Art und Dauer einer zuvor erfolgten ambulanten Antibiotikatherapie sowie eine Anamnese bezüglich einer durchgemachten COVID-19-Infektion erhoben. Es wurden darüber hinaus die erfolgten diagnostischen Mittel und deren Befunde, der klinische und intraoperative Befund, Erregernachweise, klinischer Verlauf und Komplikationen sowie die Dauer des Krankenhausaufenthalts ermittelt. Hierbei wurden die Zeiträume 2012–2021 als Zeitraum vor COVID und die Jahre 2022/2023 als Zeitraum nach COVID definiert und verschiedene Parameter zwischen beiden Gruppen verglichen.

## Ergebnisse

**Abb. 1:**
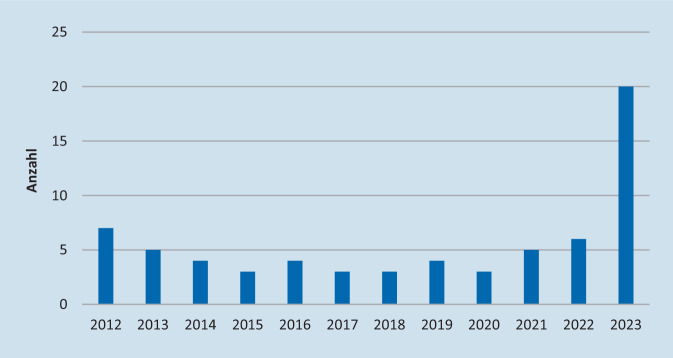
Entwicklung der Anzahl kindlicher Mastoidektomien seit 2012 in absoluten Zahlen mit deutlichem Anstieg 2022/2023 nach lange konstant bleibender Anzahl

Im untersuchten Zeitraum vom 01.01.2012 bis zum 31.06.2023 wurden insgesamt 65 Kinder bis 14 Jahre an der Klinik der Autoren mastoidektomiert. In den 10 Jahren vor COVID wurden hierbei 39 Eingriffe durchgeführt (entsprechend 3,9/Jahr), in den 2 Jahren danach 26 (entsprechend 13/Jahr). Bis 2021 waren nur sehr geringe Schwankungen in den jährlichen Inzidenzen zu sehen, mit einem Minimum von 3 und einem Maximum von 7 (Median 5). In den nur 6 in die Analyse eingeflossenen Monaten des Jahres 2023 fanden bereits 20 Mastoidektomien bei Kindern statt (Abb. 1).

**Abb. 2:**
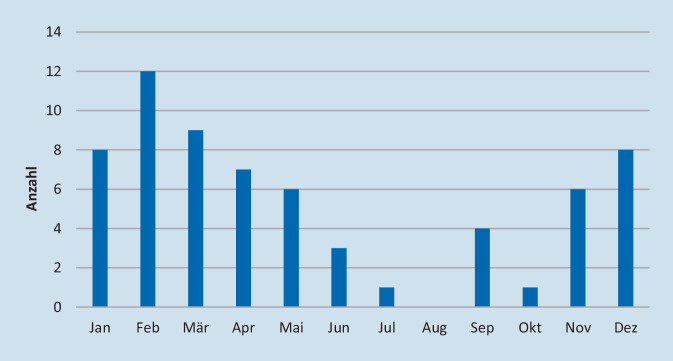
Darstellung der Häufigkeitsverteilung kindlicher Mastoidektomien im Jahresverlauf in absoluten Zahlen mit deutlicher Häufung von Dezember bis März

Es wurde eine deutliche jahreszeitliche Häufung der Erkrankungen festgestellt, mit einem Maximum in den Monaten November bis März und einem Minimum in den Sommermonaten (Abb. 2), weitgehend passend zu den jahreszeitlich sich ändernden Inzidenzen oberer Atemwegsinfektionen als häufigster Ursache einer Otitis media mit nachfolgender Mastoiditis.

**Abb. 3:**
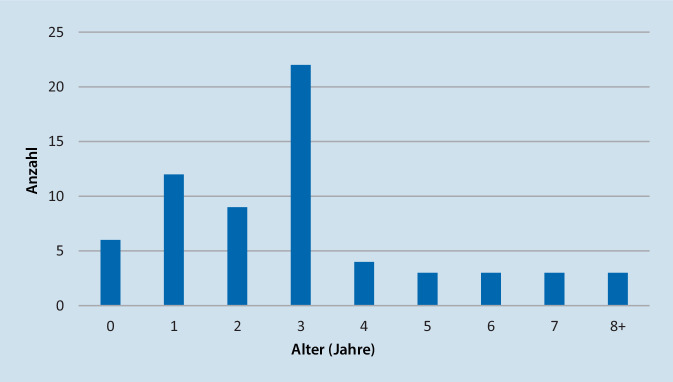
Altersverteilung der Patienten zum Zeitpunkt der Diagnosestellung in absoluten Zahlen. Deutliche Häufung bei Kindern zwischen dem 1. und 3. Lebensjahr

Hauptsächlich waren Kleinkinder im Alter von 1–3 Jahren betroffen, mit einem Maximum im dritten Lebensjahr (Abb. 3). Nur bei einem Kind bestand eine Vorerkrankung (Trisomie 21).

Otogene Beschwerden wie Otalgie, Hörminderung oder Druckgefühl bestanden im Median seit 4 Tagen (1–26 Tage) ohne signifikanten Unterschied zwischen den Zeiträumen. Eine vorherige ambulante Antibiotikatherapie hatten 19 von 65 Kindern erhalten. In der Gruppe der bis 2021 operierten Patienten erhielten 9/39 Kindern (23,08 %) eine vorherige Antibiotikatherapie, in den Jahren 2022/2023 waren dies 10/26 Kindern (38,46 %). Somit konnte hier eher ein Anstieg der antibiotisch vortherapierten Kinder beobachtet werden, dieser war jedoch nicht statistisch signifikant (*p* = 0,266).

Anamnestisch war bei 9/26 (34,62 %) der in den Jahren 2022 und 2023 therapierten Kinder eine gesicherte COVID-Infektion innerhalb der letzten 2 Jahre zu eruieren, eine akute COVID-Infektion zum Operationszeitpunkt lag bei keinem der Patienten vor.

Laborchemisch bestand bei den Kindern eine sehr variable Ausprägung einer Entzündungskonstellation, mit einem C‑reaktiven Protein (CRP) im Median von 8,48 mg/dl (0,53–27,2 mg/dl) und Leukozyten von median 13.000/µl (5100–28.600/µl). Auch die Blutsenkungsgeschwindigkeit zeigte eine große Spannbreite mit median 70 mm (15–134 mm).

Klinisch bestand bei 9/39 Kindern in der prä-COVID-Gruppe (23,08 %) eine deutliche Adenoidhyperplasie sowie ein Paukenerguss auch auf der Gegenseite bei 5/39 Patienten (12,82 %), in der Post-COVID-Gruppe waren dies 3/26 (11,54 %) respektive 15/26 Kindern (57,69 %). Diese Unterschiede erreichten bei der Adenoidhyperplasie keine statistische Signifikanz (*p* = 0,334), jedoch bei den beidseitigen Paukenergüssen (*p* < 0,001). An bei Indikationsstellung bereits bestehenden Komplikationen fanden sich eine Fazialisparese (1 ×), Schwindel als Zeichen einer Labyrinthbeteiligung (3 ×) sowie ein Gradenigo-Syndrom bei Abszedierung in der Felsenbeinspitze (1 ×). Bei allen Patienten wurde eine Computertomographie (CT) durchgeführt. Hierbei zeigte sich in 4,62 % der Fälle eine intrakranielle Abszedierung, in 18,46 % ein subperiostaler Abszess und in 30,77 % ossäre Destruktionen der Otobasis. Bei 46,15 % fand sich bildmorphologisch nur eine vollständige Verschattung des Mastoids. Eine Sinusvenenthrombose liess sich bei 6/65 Kindern diagnostizieren. Bei 9/65 Kindern erfolgte zusätzlich eine Untersuchung mittels Magnetresonanztomographie (MRT), insbesondere bei Komplikationen oder auffälligen Befunden in der CT. Hier konnte bei weiteren 4 Kindern eine in der CT nicht erkennbare intrakranielle Abszedierung nachgewiesen werden.

Bei allen Patienten wurde die Mastoidektomie sowie eine Paukendrainageneinlage der betroffenen Seite durchgeführt, bei 27,69 % war eine Paukendrainageneinlage auch auf der Gegenseite notwendig. Analog zur Adenoidhyperplasie wurden in den Prä-COVID-Jahren mehr Adenotomien gleichzeitig im selben Eingriff durchgeführt (23,08 vs. 11,54 %; *p* = 0,334). Bei allen Patienten erfolgte die Einlage eines retroaurikulären Drainageröhrchens, welches über 3 Tage mehrfach täglich abgesaugt und danach entfernt wurde.

Die mikrobiologische Diagnostik des intraoperativ gewonnenen Gewebes ergab in 30,77 % der Fälle *Streptococcus pyogenes*, in 23,08 % Pneumokokken und in jeweils 6,15 % *Haemophilus influenzae* oder sonstige Erreger, bei 33,85 % gelang kein Erregernachweis. Alle Patienten wurden empirisch i.v.-antibiotisch entweder mit Clindamycin/Cefotaxim oder mit Ampicillin/Sulbactam behandelt, eine Deeskalation der Antibiotikatherapie nach Erregernachweis war bei 22/43 Kindern mit nachgewiesenem Keim möglich. Nur bei einem Kind fand sich mit *Pseudomonas aeruginosa* ein Keim, der nicht auf die initialen Antibiotika sensibel war.

Der Krankenhausaufenthalt betrug im Median 7 Tage (3–27 Tage). Hierbei war der Krankenhausaufenthalt in der Post-COVID-Zeit signifikant länger als in der Zeit vor COVID (10,40 vs. 8,15 Tage; *p* = 0,047). Eine operative Komplikation wie Schwindel, Fazialisparese oder eine Verschlechterung des Hörvermögens im Vergleich zu präoperativ gab es im gesamten Zeitraum nicht, ebenso wenig kam es zu letalen Verläufen.

## Diskussion

Die akute Mittelohrentzündung (AOM) wird durch das Vorhandensein von Mittelohrflüssigkeit und einer Entzündung der Mittelohrschleimhaut definiert. Die akute Mastoiditis geht i. d. R. von einer akuten Otitis media aus. Frühstadien können durch Symptome der AOM wie Hörverlust, Otalgie und Fieber gekennzeichnet sein. Im weiteren Verlauf der Erkrankung kommt es zu Schwellungen, Rötungen und Druckempfindlichkeit über dem Mastoid. Die Ohrmuschel ist meist nach außen und unten verlagert. Die AOM ist eine der häufigsten Indikationen für eine Antibiotikatherapie. Entsprechend dazu wurde 2004 eine Leitlinie von der pädiatrischen Fachgesellschaft der USA zur Reduzierung der Antibiotikaverschreibung veröffentlicht, die zuletzt im Jahr 2013 überarbeitet wurde [9, 10]. Bereits 2013 wurde allerdings der Nutzen der Antibiotikatherapie gegenüber Placebo durch die Publikation von Tahtinen et al. bestätigt [11]. In Deutschland existiert derzeit lediglich eine abgelaufene Sk2-Leitlinie von 2014. Die aktuelle S2k-Leitlinie zur akuten Otitis media wird voraussichtlich bis 01.07.2025 fertiggestellt sein (https://register.awmf.org/). Die Deutsche Gesellschaft für Kinderheilkunde empfiehlt eine antibiotische Therapie bei Säuglingen unter 6 Monaten, schweren oder komplizierten AOM-Fällen (länger als 48–72 h) und relevanten Grunderkrankungen.

Die vorliegenden Daten zeigen einen abweichenden Trend auf. Im Vergleich zu früheren Studien zeigt diese Kohorte, dass viele der Patienten der Autoren bereits vor der Mastoiditis antibiotisch behandelt wurden. Auch dieser Trend verstärkte sich im Verlauf der Zeit, so stieg der Anteil der vortherapierten Kinder von 23 % bis zum Jahr 2021 auf fast 39 % in den Jahren 2022/2023 an, obwohl ein allgemeiner Rückgang der Antibiotikaverordnungen zu verzeichnen sein müsste. Der beobachtete Anstieg der Mastoidektomien im Rahmen der AOM in den Jahren nach der COVID-Pandemie gibt einen weiteren Anlass zur Diskussion. Insbesondere die höhere Zahl vorbehandelter Kinder in dieser Kohorte könnte auf eine Verschlechterung oder Veränderung des Schweregrads der Fälle hinweisen. Wenngleich dieser Anstieg statistisch nicht signifikant war, ist es dennoch wichtig, die möglichen Auswirkungen der COVID-Pandemie auf den Schweregrad der Mastoiditisfälle zu berücksichtigen und weiter zu erforschen.

Ende 2022 wurde von der Weltgesundheitsorganisation (WHO) ein Statement über eine länderübergreifende Zunahme von Infektionen mit invasiven Streptokokken der Gruppe A veröffentlicht (https://www.who.int/emergencies/disease-outbreak-news). In England wurden im Rahmen einer Studie von 01/2018 bis 11/2022 ein deutlicher Anstieg der Infektionen mit invasiven Streptokokken der Gruppe A, insbesondere bei Kindern unter 15 Jahren, im Jahr 2022 verzeichnet [12]. Bemerkenswert ist, dass fast 35 % der Patienten aus der Kohorte der Autoren aus dem Jahr 2022 eine vorangegangene SARS-CoV-2-Infektion innerhalb der letzten 2 Jahre hatten. Es ist bekannt, dass SARS-CoV‑2 im Vergleich zu anderen viral assoziierten Atemwegsinfektionen eine stärkere und länger anhaltende zelluläre Reaktion auslöst, selbst bei leichtgradigen oder asymptomatischen Verläufen [13]. Die Frage, ob eine SARS-CoV-2-Infektion die Abwehrfähigkeit des Immunsystems der Kinder beeinflusst, ist relevant und wird durch klinische Daten unterstützt. Von 01/2022 bis 05/2022 gab es z. B. 232 Fälle von schwerer akuter Hepatitis unbekannter Ursache bei Kindern unter 16 Jahren, wobei einige dieser Fälle mit SARS-CoV-2-Infektionen (akut oder in den vergangenen Monaten) in Verbindung gebracht wurden [13].

Der deutliche Anstieg der Mastoidektomien seit Beginn der COVID-Pandemie erfordert eine detaillierte Untersuchung möglicher Zusammenhänge. Die saisonale Häufung mit einem Maximum in den Wintermonaten steht im Einklang mit der Inzidenz von Infektionen der oberen Atemwege. Die hohe Rate der Mastoidektomien könnte sich auf den ersten Blick theoretisch durch den Anstieg der Atemwegsinfektionen erklären. Der Anstieg der Atemwegsinfektionen wurde in der Studie von Maison et al. berichtet. Die Studie beobachtete einen 4fachen Anstieg der Atemwegsinfektionen im Jahr 2022 im Vergleich zu 2019 [14]. Diese Zunahme könnte auch den Anstieg von AOM und damit verbundenen Mastoiditisfälle erklären. Im Patientenkollektiv der Autoren blieb die Anzahl der Mastoidektomien im Jahr 2022 nahezu vergleichbar mit der von 2012. Für andere obere Atemwegserkrankungen zeigt sich ein ähnliches Bild, trotz bisher nur spärlicher Literatur hierzu. So konnte ein Anstieg sowohl der Häufigkeit von Sinusitiden als auch ein Anstieg der damit verbundenen komplikativen Verläufe in den USA nach dem Ende der Pandemie aufgezeigt werden.

Die Diagnostik der Mastoiditis ist dagegen weit weniger kontrovers. Die Ohrmikroskopie und die klinische Untersuchung sind nach wie vor gut geeignet, um eine Mastoiditis und eventuelle ursächliche Faktoren wie Adenoide identifizieren zu können. Diesbezüglich bestand in diesem Kollektiv nur bei 12 % der Patienten auch eine Adenoidhyperplasie, dennoch fanden sich sehr hohe Raten an Paukenergüssen auch auf der Gegenseite in der Post-COVID-Gruppe. Eine ursächliche Erklärung hierzu liess sich in den Datenanalysen jedoch nicht finden. Die Meinungen über bildgebende Verfahren hingegen gehen in der Literatur auseinander. Einige Autoren machen ihre Entscheidung hinsichtlich einer Bildgebung von der Unterscheidung zwischen komplizierter und unkomplizierter Mastoiditis abhängig, während andere die Durchführung einer präoperativen Schnittbildgebung für unerlässlich halten [5]. Nach Meinung der Autoren ist eine präoperative Schnittbildgebung unerlässlich, um klinisch nicht offensichtliche Komplikationen rechtzeitig zu erkennen und eine angemessene Therapievorbereitung zu ermöglichen.

*Streptococcus pneumoniae* wurde in mehreren Studien als Hauptkeim für die akute Mastoiditis bei Kindern identifiziert [15, 16]. Laulajainen-Hongisto analysierte das bakterielle Spektrum von 56 Kindern mit akuter Mastoiditis und stellte fest, dass *Streptococcus pneumoniae* in 38 % der Fälle nachgewiesen wurde, gefolgt von *Streptococcus pyogenes* und *Pseudomonas aeruginosa* mit jeweils 11 % der Fälle [15]. In dem hier vorliegenden Kollektiv wurden die meisten Infektionen durch Streptokokken verursacht. Die Divergenz zu den anderen Studien könnte durch die Pneumokokkenimpfung erklärt werden, die die STIKO für alle Säuglinge ab dem Alter von 2 Monaten empfiehlt (https://www.rki.de/). Jedoch hat sich die Inzidenz der akuten Mastoiditis vor und nach Einführung der Impfung im Jahr 2000 dadurch nicht wesentlich verändert [17].

Zu den Komplikationen der Mastoidektomie gehören beispielsweise Blutungen, Hirnverletzungen, Verletzungen des Gesichtsnervs, Taubheit und Schwindel. In dem Kollektiv der Autoren traten keine Operationskomplikationen auf, die einen Revisionseingriff oder einer weiteren Intervention erforderlich gemacht hätten. Es gab einen Unterschied in Bezug auf den Krankenhausaufenthalt, welcher im Median 7 Tage betrug. Dieser war in der Post-COVID-Zeit signifikant länger.

Als Fazit bleibt zu konstatieren, dass die Ursache der signifikanten Zunahme an kindlichen Mastoidektomien nicht abschließend aus diesen retrospektiven Daten geschlussfolgert werden kann. Ein verändertes Verschreibungsverhalten von Antibiotika kann anhand der Daten nicht belegt werden, eher fand sich hier eine Zunahme an vortherapierten Kindern in den letzten Jahren. Eine Assoziation zu einer vorangegangenen COVID-Infektion erscheint aufgrund des zeitlichen Verlaufs plausibel, ist jedoch letztlich hiermit nicht beweisbar. Die Auswertungen der Inzidenzen folgender Jahre wird zeigen, inwiefern es sich um ein zeitlich begrenztes Phänomen handelt.

## Fazit für die Praxis

- Die Mastoiditis des Kindes sollte stationär therapiert werden.
- Bei Mastoiditis ohne Komplikationen ist ein konservativer Therapieversuch gerechtfertigt.
- Bei klinischer Verschlechterung oder ausbleibender Besserung sowie bei Verlauf mit Komplikationen ist eine Mastoidektomie mit Einlage einer Paukendrainage indiziert.
- Als Komplikationen gelten subperiostale oder intrakranielle Abszedierung, Innenohrbeteiligung, Fazialisparese, Sinusvenenthrombose, Meningitis und neurologische Ausfälle.
- Eine als ursächlich angesehen Adenoidhyperplasie sollte speziell bei Kindern unter 6 Jahren im selben Eingriff saniert werden.

## References

[1] Jamal A, Alsabea A, Tarakmeh M et al (2022) Etiology, diagnosis, complications, and management of acute otitis media in children. Cureus 14:e28019. 10.7759/cureus.28019 36134092 10.7759/cureus.28019 PMC9471510

[2] Tamir SO, Bialasiewicz S, Brennan-Jones CG et al (2023) ISOM 2023 research panel 4—diagnostics and microbiology of otitis media. Int J Pediatr Otorhinolaryngol 174:111741. 10.1016/j.ijporl.2023.111741 37788516 10.1016/j.ijporl.2023.111741

[3] House HP (1946) Otitis media—a comparative study of the results obtained in therapy before and after the Introduction of the sulfonamide compounds. Arch Otolaryngol 43:371–378 10.1001/archotol.1946.00680050387005 21028193

[4] Groth A, Enoksson F, Hultcrantz M et al (2012) Acute mastoiditis in children aged 0–16 years—a national study of 678 cases in Sweden comparing different age groups. Int J Pediatr Otorhinolaryngol 76:1494–1500. 10.1016/j.ijporl.2012.07.002 22832239 10.1016/j.ijporl.2012.07.002

[5] Cassano P, Ciprandi G, Passali D (2020) Acute mastoiditis in children. Acta Biomed 91:54–59. 10.23750/abm.v91i1-S.9259 32073562 10.23750/abm.v91i1-S.9259 PMC7947742

[6] Tanislav C, Kostev K (2022) Fewer non-COVID-19 respiratory tract infections and gastrointestinal infections during the COVID-19 pandemic. J Med Virol 94:298–302. 10.1002/jmv.27321 34491581 10.1002/jmv.27321 PMC8661971

[7] Kuitunen I, Artama M, Mäkelä L et al (2020) Effect of social distancing due to the COVID-19 pandemic on the incidence of viral respiratory tract infections in children in finland during early. Pediatr Infect Dis J 39:E423–E427. 10.1097/Inf.0000000000002845 32773660 10.1097/INF.0000000000002845

[8] Kruizinga MD, Noordzij JG, van Houten MA et al (2023) Effect of lockdowns on the epidemiology of pediatric respiratory disease—a retrospective analysis of the 2021 summer epidemic. Pediatr Pulmonol 58:1229–1236. 10.1002/ppul.26327 36695757 10.1002/ppul.26327

[9] Lieberthal AS, Ganiats TG, Cox EO et al (2004) Diagnosis and management of acute otitis media. Pediatrics 113:1451–1465 15121972 10.1542/peds.113.5.1451

[10] Hoberman A, Paradise JL, Rockette HE et al (2011) Treatment of acute otitis media in children under 2 years of age. New Engl J Med 364:105–115. 10.1056/NEJMoa0912254 21226576 10.1056/NEJMoa0912254 PMC3042231

[11] Tähtinen PA, Laine MK, Huovinen P et al (2011) A placebo-controlled trial of antimicrobial treatment for acute otitis media. New Engl J Med 364:116–126. 10.1056/NEJMoa1007174 21226577 10.1056/NEJMoa1007174

[12] Guy R, Henderson KL, Coelho J et al (2023) Increase in invasive group A streptococcal infection notifications, England, 2022. Euro Surveill 28:14–19. 10.2807/1560-7917.Es.2023.28.1.2200942 10.2807/1560-7917.ES.2023.28.1.2200942 PMC9817207 36695450

[13] Rotulo GA, Palma P (2023) Understanding COVID-19 in children: immune determinants and post-infection conditions. Pediatr Res. 10.1038/s41390-023-02549-7 36879079 10.1038/s41390-023-02549-7 PMC9987407

[14] Maison N, Omony J, Rinderknecht S et al (2023) Old foes following news ways?-pandemic-related changes in the epidemiology of viral respiratory tract infections. Infection. 10.1007/s15010-023-02085-w 37644253 10.1007/s15010-023-02085-w PMC10811157

[15] Laulajainen-Hongisto A, Saat R, Lempinen L et al (2014) Bacteriology in relation to clinical findings and treatment of acute mastoiditis in children. Int J Pediatr Otorhinolaryngol 78:2072–2078. 10.1016/j.ijporl.2014.09.007 25281339 10.1016/j.ijporl.2014.09.007

[16] Bilavsky E, Yarden-Bilavsky H, Samra Z et al (2009) Clinical, laboratory, and microbiological differences between children with simple or complicated mastoiditis. Int J Pediatr Otorhinolaryngol 73:1270–1273. 10.1016/j.ijporl.2009.05.019 19539381 10.1016/j.ijporl.2009.05.019

[17] Choi SS, Lander L (2011) Pediatric Acute Mastoiditis in the Post-Pneumococcal Conjugate Vaccine Era. Laryngoscope 121:1072–1080. 10.1002/lary.21727 21520127 10.1002/lary.21727

